# Commentary: Wogonoside alleviates metabolic dysfunction-associated steatohepatitis by modulating AMPK and xCT/GPX4 pathways to restrain lipid dysregulation and ferroptosis

**DOI:** 10.3389/fphar.2026.1965553

**Published:** 2026-10-21

**Authors:** Yuchen Fang, Lianjun Xing

**Affiliations:** Department II of Digestive Diseases, Longhua Hospital, Shanghai University of Traditional Chinese Medicine, Shanghai, China

## Introduction

Metabolic dysfunction-associated steatohepatitis (MASH) is characterized by hepatic steatosis, hepatocellular injury and inflammation, while fibrosis staging provides additional prognostic information (EASL-EASD-EASO, 2024). Wogonoside (WOG), a flavonoid from *Scutellaria baicalensis*, has been reported to ameliorate liver injury, dyslipidemia, inflammation and oxidative stress in mice with nonalcoholic fatty liver disease (NAFLD) (Jiang et al., 2020), providing a rationale for further investigation.

We read the study with interest by Wang et al. (2026), which examined WOG in high-fat and high-fructose diet (HFFD)-fed mice and free fatty acid (FFA)-treated HepG2 cells. Its integration of metabolic phenotyping, histology, transcriptomics and erastin/ferrostatin-1 experiments provides valuable preclinical evidence. The authors proposed that WOG alleviates MASH partly by suppressing ferroptosis through the xCT/GPX4 axis, based on reduced lipid peroxidation and Fe^2+^ accumulation alongside increased xCT and GPX4 expression. Ferroptosis is a form of regulated cell death driven by iron-dependent lipid peroxidation (Mishima et al., 2025). However, this study shows evidence that WOG attenuates ferroptosis-associated biochemical changes, but does not establish that WOG provides protection against ferroptosis. We would like to highlight several points that may help to refine the interpretation of the ferroptosis findings, further characterize the histological response, and improve the clarity of the report.

### Interpreting the ferroptosis evidence

All figures mentioned below, including supplementary figures, refer to the results published in the original study by Wang et al. (2026). In Figure 7, WOG treatment under FFA + erastin challenge reduced lipid peroxide (LPO) levels (Figure 7B) and Fe^2+^ levels (Figure 7D), and increased xCT and GPX4 expression (Figures 7N and 7O). Together, these results provide meaningful evidence that WOG attenuates biochemical changes closely associated with ferroptosis.

Ferroptosis is, however, a form of regulated cell death. Lipid peroxidation, iron accumulation, glutathione depletion, and altered xCT/GPX4 expression are important mechanistic indicators, but they do not independently establish that ferroptosis occurred. Recent expert recommendations emphasize convergent evidence, including cell-death assessment, rescue by ferroptosis inhibitors, measurement of lipid peroxidation and evaluation of alternative cell-death pathways (Mishima et al., 2025). This study did not report viability or death under the FFA + erastin challenge or assess whether WOG or Fer-1 rescued any resulting cell death. Supplementary Figure S1 evaluates WOG’s cytotoxicity at the selected concentrations, but does not establish whether FFA or erastin induced death. Thus, the current findings support attenuation of a ferroptosis-associated biochemical phenotype, while protection against ferroptosis remains to be established.

One way to validate this finding is to combine the existing Liperfluo assay, or an alternative probe such as C11-BODIPY 581/591, with propidium iodide (PI)- or SYTOX-based cell-death measurements and a complementary viability endpoint. Assessing whether WOG rescues measured death, alongside Fer-1 and an additional inhibitor such as liproxstatin-1, would further support that WOG directly inhibits ferroptosis.

In Figure 7H, the bracketed comparison appears to indicate lower LPO levels in the FFA + Fer-1 + WOG group than in the FFA + Fer-1 group. This warrants clarification given the text’s description of a “similar, non-additive effect”. In addition, the figure legend defines asterisks as comparisons with the FFA group, whereas the brackets appear to indicate other pairwise comparisons. Clarifying these comparisons and the basis for the non-additive interpretation would improve the consistency between the figure and text.

### Histological assessment of MASH

The HFFD model reproduced metabolic and hepatic abnormalities relevant to MASH. H&E and Oil Red O images support steatosis, while TNF-α immunohistochemistry supports inflammatory signaling. However, no standardized NAFLD Activity Score (NAS), Steatosis, Activity and Fibrosis (SAF) component scores or blinded pathological evaluation were reported. TNF-α staining cannot substitute for assessment of lobular inflammation, and hepatocellular ballooning was not quantified. Blinded assessment of steatosis, lobular inflammation and ballooning, using criteria appropriate to the experimental model, would better define disease activity and treatment response (Kleiner et al., 2005). Activity scores should support, rather than replace, morphological evaluation of steatohepatitis.

Neither collagen-specific staining, such as Sirius Red or Masson’s trichrome, nor fibrosis staging was reported. Assessing the presence and extent of fibrosis would help characterize the model and clarify the scope of WOG’s effects. However, HFFD models may develop limited fibrosis, and collagen staining might reveal only mild changes. Both the absence of fibrosis assessment and the model’s potential limitations should therefore be considered when evaluating the evidence for WOG’s antifibrotic effects.

### Transcriptomic and reporting clarifications

A few numerical discrepancies appear to warrant clarification. Figures 3B and 3C indicate differentially expressed gene (DEG) totals of 535 (333 + 202) and 335 (162 + 173), whereas the text and Figure 3D indicate 540 and 381, respectively. The intersection is reported as 129 genes in the text but 138 in Figure 3D. The most straightforward explanation is a reporting or typographical error, if so, this would not affect the underlying analyses. A more consequential possibility is that filtering or annotation inconsistencies affected the input gene lists, potentially altering overlap or enrichment results and their mechanistic interpretation. Reconciling the counts, input gene lists and selection criteria for each analysis would clarify whether correction of the presentation alone is sufficient.

To support the description of overlapping DEGs as “normalized” by WOG, directional reversal should be demonstrated; overlap alone is insufficient. Using nominal *P* < 0.05 with |log_2_FC| > 1 can provide a pragmatic screen for exploratory DEG analysis. These thresholds do not, however, control the gene-level false discovery rate. We therefore regard the resulting transcriptomic findings as hypothesis-generating evidence that can guide subsequent mechanistic validation.

## Discussion

Wang et al. provide valuable preclinical evidence of WOG-associated metabolic and hepatic protection in experimental MASH. Recent studies illustrate complementary approaches that could extend these findings. Peleman et al. (2024) assessed cell death alongside lipid peroxidation, while Yin et al. (2025) measured viability and LDH release in lipid-challenged hepatocytes. Incorporating comparable endpoints with ferroptosis-inhibitor rescue would help establish whether WOG’s biochemical effects translate into protection against ferroptosis. Building on the existing histological analyses, standardized activity scoring and fibrosis assessment could further define WOG’s benefits. Yin et al. (2025), for example, complemented NAS with quantitative assessment of collagen deposition. These complementary assessments, together with clearer transcriptomic reporting, would strengthen the foundation established by Wang et al. for further investigation of WOG in MASH.

Overall, this study contributes meaningful evidence supporting WOG as a promising candidate for further investigation in MASH. The conclusions would be further strengthened by direct assessment of ferroptosis, standardized histological scoring, and clarification of the reporting discrepancies.
